# Anti-Thyroid Peroxidase Antibody in Vitiligo: A Prevalence Study

**DOI:** 10.1155/2015/192736

**Published:** 2015-01-11

**Authors:** R. Dash, A. Mohapatra, B. S. Manjunathswamy

**Affiliations:** ^1^Department of Dermatology, Venereology and Leprosy, Jawaharlal Nehru Medical College and Dr. Prabhakar Kore KLES Hospital, Belgaum, Karnataka 590010, India; ^2^The INCLEN Trust International, New Delhi 110020, India

## Abstract

*Aim.* The aim of the study was to study the relation of vitiligo with demographic data like age, sex, and duration and determine the prevalence of thyroid autoimmunity in vitiligo patients. *Materials and Methods.* This study was a cross sectional study consisting of 100 patients clinically diagnosed (old and new) as having vitiligo irrespective of age or sex. Patients with known thyroid disease on supplementation therapy, or who had undergone thyroid surgery, those on antithyroid medication, patients with other causes of leukoderma, and cases who do not provide informed consent were excluded from the study. Serum TSH and anti-TPO antibodies were measured in all the patients. *Results.* The prevalence of anti-TPO antibody positivity was found to be 28%. *Conclusion.* According to our study, none of our vitiligo patients had symptoms or signs of thyroid disease at the time of presentation but, on biochemical evaluation, anti-TPO antibodies were found in a considerable number of patients. Hence, we recommend screening of these patients with thyroid antibodies.

## 1. Introduction

Vitiligo is defined as a specific, common, often heritable, and acquired dermatological disorder characterized by well-circumscribed, milky-white cutaneous macules and patches devoid of identifiable melanocytes [1]. Vitiligo affects 0.1–2% of the world's population. It usually begins in childhood or adolescence, with peak onset at 10 to 30 years, but it may occur at any age. Both sexes are equally affected [2]. Clinically it can be broadly classified into three major clinical types, segmental, nonsegmental, and mixed (segmental and nonsegmental) [3]. It is believed that vitiligo is a multifactorial polygenic disorder with a complex pathogenesis. Although several theories (that include autoimmune, autocytotoxic, biochemical, neural, and genetic mechanisms) have been proposed to explain the loss of epidermal melanocytes in vitiligo, the precise cause remains unknown [2]. At present, the autoimmune theory is most plausible [4]. Over the years there have been various reports of associations between vitiligo and other autoimmune diseases notably thyroid disease and antithyroid antibodies [5–7]. These autoimmune thyroid diseases are characterized by elevated serum antibodies directed against thyroid-specific antigens like thyroid peroxidase (TPO) and thyroglobulin (Tg). A mean prevalence of 20.8% has been reported in patients with vitiligo for thyroid-specific autoantibodies [8]. In India, a prevalence of 31.4% has been reported for the same [9]. On reviewing the literature we could not find many similar studies in this part of India; hence we were prompted to undertake this study with the objectives mentioned in the following section. Therefore, we undertook this study to look into the characteristics of vitiligo patients with thyroid autoimmunity as indicated by their anti-thyroid peroxidase antibody (anti-TPO Ab) titres.

## 2. Objectives

The study was undertaken with the following objectives:

1. to describe the clinicodemographic profiles of the study participants with vitiligo;
2. to evaluate the prevalence of thyroid autoimmunity (measured by anti-TPO Ab titres) in the study participants with vitiligo;
3. to compare the clinicodemographic profiles of the study participants according to their thyroid autoimmunity status.

## 3. Material and Methods

### 3.1. Study Design

A cross-sectional study design is suited for estimating prevalence of disease and traits. It can be used to describe participants' attributes as well. To compare individuals with the disease/trait and those without, we adopted this analytical cross-sectional design for this study.

### 3.2. Study Site

The study was conducted in the Dermatology Out-Patient Department of a tertiary care hospital in Karnataka.

### 3.3. Study Duration

The study synopsis was submitted to the Institutional Review Committee in 2011 following which data collection was undertaken in 2012.

### 3.4. Ethics Clearance

The Institutional Ethics Committee on Human Subjects Research, 2011-12, granted approval, subsequent to which data collection for the study was initiated.

### 3.5. Sampling Frame

Patients attending the Dermatology OPD of the tertiary care hospital in Karnataka (anonymized study site) between January and December 2012, who were clinically diagnosed (old and new) as having vitiligo and willing to provide written informed consent, were decided to be considered for inclusion in the study (inclusion criteria).

### 3.6. Selection Criteria

Patients with known thyroid disease, or on thyroid supplementation therapy or antithyroid medication, those who had undergone thyroid surgery, patients with other causes of leukoderma, and those who did not provide informed consent were excluded from the study. The inclusion criteria have been mentioned above.

### 3.7. Sample Size

Considering the formula, *N* = (*z* ^2^ × *p* × *q*)*d* ^2^ = 4*pq*/*d* ^2^, where *p* (prevalence) = 31.4% [9]; *q* (100 − *p*) = 100 − 31.4 = 68.6%; *d* (absolute error) = 10%; *z* (95% confidence level) = 1.96, that is, ~2.

Consider *N* = 4 × 31.4 × 68.6/(10 × 10) = 86.16, that is, ~87 (rounded to the next higher integer).

We included additional 13 (~15%) participants to avoid data insufficiency due to a higher sample size requirement in the event of a lower prevalence reported from this study or any inadvertent data loss during the study conduct. Thus, the total sample size for the study was 100.

### 3.8. Sampling Techniques

Consecutive patients satisfying the selection criteria were included in study till the requisite sample size was met.

### 3.9. Data Collection

A structured proforma was designed to collect information from the study participants. This proforma was pretested in the Dermatology OPD (where the study was later conducted) and finalised. Detailed medical history taking and physical examination were undertaken by the primary researcher under guidance of the mentor to make a clinical diagnosis of vitiligo, and the same were documented in the proforma. Venous blood was collected from the subjects and transported to the hospital's central laboratory where it was processed to assess serum thyroid stimulating hormone (TSH) and anti-thyroid peroxidase antibodies (anti-TPO Ab) levels. Serum TSH was quantitatively measured by quantitative chemiluminescent immunoassay using a commercially available kit on ADVIA Centaur XP (Siemens). A diagnosis of “hypothyroidism” was made when the thyroid function test showed raised serum TSH levels, and “hyperthyroidism” was diagnosed if depressed levels of TSH were detected (normal range of serum TSH being 0.49–4.67 *μ*IU/mL). Anti-TPO Ab was assayed by quantitative chemiluminescent immunoassay on ADVIA Immulite 1000 (Siemens). The serum was considered “positive” for thyroid autoimmunity if the concentration was greater than 35 IU/mL (normal range: 0.00–35 IU/mL was labelled “negative”). The cut-offs were decided as per the mandate of the central laboratory which has been finalized by the concerned medical specialities.

### 3.10. Data Management (Extraction, Cleaning, and Analysis)

Data from the proforma was extracted and entered into Microsoft Office 2013 Excel spreadsheet. Range and dimensions of the study variables and data were checked during data cleaning. Detailed profiling (descriptive and comparative) of the study participants vis à vis their anti-TPO antibody status was done using SPSS version 16.0. As per indication, nonparametric tests (Mann Whitney *U* test, Chi-squared test, and Fisher's exact probability test) were applied to test statistical significance at *P* < 0.05.

### 3.11. Representation of Data

Data was represented in contingency tables in the form of absolute numbers and proportions. Results from tests for significance were enumerated in the tables and graphs, wherever applicable.

## 4. Results

A total of 100 patients with vitiligo who satisfied the selection criteria were included in the study. The demographic characteristics of the study participants are described in Table 1.

The age of the participants ranged from 2 to 62 years. The mean age of males was 33.88 + 15.86 years (median = 35 years) while that of females was 26.44 ± 13.70 years (median = 24 years). The mean ± standard deviations for the serum concentrations of TSH and anti-TPO Ab were 5.09 + 21.87 IU and 75.41 + 206.77 IU, respectively. The correlation between the two (Spearman's rho = 0.052) was not statistically significant (*P* = 0.609). Only two (7.1%) of the anti-TPO Ab positive participants and one (1.4%) of the negative participants had hyperthyroidism. Similarly, only five (17.9%) in the former and eight (11.1%) in the latter were hypothyroid. No significant association was found between the anti-TPO antibody status and the thyroid function status (euthyroid versus thyroid dysfunction) of the study participants (*P* = 0.126).

The prevalence of thyroid autoimmunity (defined as serum anti-TPO Ab titre >35 IU/mL) was found to be 28.0 (19.2–36.8)%.

The comparative characteristics of the anti-TPO Ab positive and negative participants are given in Table 2.

A significant difference was observed between the mean age of participants with anti-TPO Ab positivity and those with negative status, the former being more than the latter. A history of vitiligo precipitating “trigger” was elicited more commonly with those with anti-TPO Ab as compared to those with anti-TPO Ab negative (*P* = 0.037). Physical trauma was the most common trigger of vitiligo in the anti-TPO Ab positive participants. Autoimmune disease history was found in two of the 28 patients who tested positive for anti-TPO Ab. One had duodenitis/gastritis with pernicious anaemia and the other alopecia areata. None of anti-TPO Ab negative participants provided a positive history for autoimmune diseases. No significant association could be elicited for history of autoimmune diseases with anti-TPO Ab status (*P* = 0.076). A total of three patients with anti-TPO antibody positivity had a family of autoimmune diseases (one thyroid and two diabetes mellitus).

## 5. Discussion

### 5.1. Clinicodemographic Profile of Study Participants with Vitiligo

#### 5.1.1. Age and Sex of the Participants

In our study, female participants formed the majority (59%) and presented at an earlier age as compared to the males. Earlier studies have suggested that vitiligo being an autoimmune disease could be more common in females [5, 16–18]. Moreover, since vitiligo causes cosmetic aberrations, it could be generating higher concern among females, leading to an increased and earlier attendance of females in the Dermatology OPD.

#### 5.1.2. Reported Age at Onset and Duration of Vitiligo

The mean participant reported age at onset in our study was 24.80 ± 0.16 years (median: 21.25 years; range: 1.67–63 years). Other investigators have also reported that majority of vitiligo cases have an onset in and around 2nd and 3rd decade of life [17–20]. However, a study in India reported a later onset of the disease, with a mean age of 55 years [21]. These data reinforce that vitiligo is a disease that can occur at any age. In our study, females manifested vitiligo around 10 years earlier than males (*P* = 0.004). This trend was opposite to that noted by Majumder et al. [19] who reported that males had a seven-year early onset of vitiligo than females. Other researchers have found no significant difference in age at onset between the two genders [22, 23].

The mean duration of vitiligo in our study participants was 4.64 ± 6.05 (median = 2) years (no male-female difference) which is close to the finding of another Indian study (3.7 years) [9].

#### 5.1.3. Family History of Vitiligo

A positive family history of vitiligo was found in 13% of the participants which is similar to the findings by Hann et al. and lower than others [11, 17, 19, 24]. A lower familial aggregation in our study could be possible because it was reported by participants' recall.

#### 5.1.4. History of Precipitating Factors

We probed to find out if the participants could recollect any particular factor that precipitated vitiligo in them for the first time. In our study while majority (92%) of them did not relate to the incidence of vitiligo lesions with any precipitating factor, we found history of emotional stress in two percent of participants; three percent reported that the vitiligo lesions in them manifested at sites of trauma. While one percent attributed vitiligo precipitation to physical stress, another reported sun exposure as the triggering factor for vitiligo lesions. One of the females recollected the first appearance of vitiligo during pregnancy.

#### 5.1.5. Disease Activity [25]

A low VIDA score (six-point scale) indicates less activity. Majority of the participants reported relatively low disease activity. We could not find relevant literature that has looked into VIDA scores in vitiligo patients in a contextually relevant setting.

#### 5.1.6. Types of Vitiligo

In our study, segmental vitiligo was found in only 4% of the cases. The reported prevalence in the literature of segmental vitiligo ranges from 3.5% to 20.5% of all patients with vitiligo [26]. Among nonsegmental vitiligo, vitiligo vulgaris was the commonest (71%) type of vitiligo in our study. Other investigators have also found vitiligo vulgaris to be the commonest type of vitiligo [11, 24, 27].

#### 5.1.7. Site of Onset and Current Site of Involvement of Vitiligo

Evidence suggests that the primary site of involvement in vitiligo is sun-exposed areas [21, 27, 28]. A study from Kuwait [29] noted that majority of their patients reported depigmentation in lower limbs followed by upper limbs, head and neck, trunk, and mucosae in decreasing order. In our study also majority of the subjects, that is, 34%, had lower limbs as the site of onset followed by head and neck, trunk (22% each), upper limbs (20%), and genitals (2%). The most common site of current involvement in our study population was lower limb (75%) followed by upper limb (52%), head and neck (42%), trunk (42%), mucosa (17%), and genitals (9%).

#### 5.1.8. Vitiligo Area Severity Index (VASI) [25]

Most of our participants (60%) reported VASI between the ranges of 1.00 and 4.99. This should be the usual presentation of vitiligo cases at the dermatology OPD. It could indicate that patients with a score of “<1” usually do not seek health care. Simultaneously, a higher VASI could reflect negligence for the lesions or rapid progression. We could not find other studies which have assessed VASI on vitiligo patients but the prevalence of body surface area involved (calculated by rule of nines) was seen to vary from 5 to 50% in various studies [14, 28].

### 5.2. Prevalence of Thyroid Autoimmunity in the Study Sample

The upper limit for anti-TPO Ab was as per the hospital laboratory reference range. We defined an anti-TPO antibody serum titre of ≥35 IU/mL as “*anti-TPO Ab positive*” and evaluated the same in these 100 participants. This was used as a proxy indicator for thyroid autoimmunity in the study participants. Twenty-eight of the 100 participants had a positive titre. Thus, the sample prevalence of anti-TPO antibody positivity (thyroid autoimmunity) was estimated at 28% (95% CI for population prevalence: 19.2–36.8%). Previous researchers have reported prevalence of anti-TPO antibody positivity in vitiligo patients from around 11% to as much as 50% (Table 3).

The estimated prevalence in our study lies within the reported range in the available literature. However, we should bear in mind that demographic characteristics and health profile of the participants in studies discussed play determining roles in the prevalence estimates. Care seeking behaviour of the OPD attendees and willingness to participate in the study also affect the estimation of prevalence. Moreover, hospital recruited samples may not be true representatives of the general population and could underestimate disease prevalence if the care seeking behaviour is poor. Nevertheless, with our study site being a popular health care setup in the region, we believe that the sampled participants could provide an approximate population estimate of the catchment area for diseases like vitiligo where the patient usually seeks medical advice. However, this again could be an underestimation in the presence of other healthcare providers in the region.

### 5.3. Clinicodemographic Profiles of the Study Participants according to Their Thyroid Autoimmunity Status

#### 5.3.1. Age and Sex Distribution of Participants

The mean age of the anti-TPO antibody positive participants was 34.5 years as compared to 27.5 years in the antibody negative participants (*P* = 0.037). Participants in the anti-TPO Ab positive group were mostly in their third decade and beyond while those found negative were mostly in the second decade of life and their representation consistently decreased in the subsequent decadal age bands. Both Mandry et al. and Nunes have reported that patients with autoantibodies tend to have a higher mean age when compared to patients without autoantibodies which is in accordance to our findings [13, 28].

In the currently discussed study, the proportion of females in the anti-TPO Ab positive group was higher (71.4%) than in the anti-TPO Ab negative group (54.2%), with the female : male ratio in the two groups being 5 : 2 and ~5 : 4, respectively. However, sex predilection for females in either group was not statistically significant (*P* = 0.115). Both Altaf et al. and Nunes observed a significantly higher incidence of thyroid dysfunction as well as anti-TPO Ab positivity in females in their respective studies [15, 28]. Daneshpazhooh et al. reinforced this finding as they noted anti-TPO Ab to be significantly more common in female patients especially in young women in the age range of 18 to 35 years, compared with control group [14].

#### 5.3.2. Reported Age at Onset of Vitiligo in Participants and Its Correlation with Their Gender

We did not find a statistically significant difference in the reported mean age at onset of vitiligo according to anti-TPO Ab status. Earlier investigators have reported that patients with autoantibodies have a later onset of disease when compared to patients without autoantibodies [13, 28]. However recently, Altaf et al. found that a significant number (86%) of patients with anti-TPO antibody positivity had early onset vitiligo [15]. Dave and colleagues also found earlier age at onset to be associated with thyroid abnormality but without statistical significance [9].

#### 5.3.3. Duration of Vitiligo

Betterle showed a significant relationship between anti-TPO Ab with long lasting vitiligo in their patients [10]. In our study, “positive” participants did report a longer duration of vitiligo but did not differ significantly from the “negative” ones.

#### 5.3.4. Family History of Vitiligo in Participants

History of vitiligo in the family was reported by 10.7% of our anti-TPO positive patients while it was 13.9% in anti-TPO negative patients. We enquired if history of vitiligo in the family was more commonly elicited in either of the two participant groups but found no such association. Family history of vitiligo was seen in 5% of the cases with thyroid abnormality in the study by Dave et al. [9].

#### 5.3.5. History of Precipitating (Trigger) Factors

Emotional stress has been mentioned as a triggering factor for vitiligo by researchers, but these data are still limited and there is no established evidence in the literature [30]. In some studies, history of a precipitating factor (physical injury being most common that is Koebner's phenomenon followed by emotional stress, sunburn, systemic illness, pregnancy, and parturition) has been shown to be present in 10–76% of cases, but the high and sometimes concomitant occurrence of these factors as such makes it difficult to assess the exact role played by them in precipitating vitiligo [31]. Certainly, evidence looking into association of history of trigger precipitated vitiligo with thyroid autoimmunity is lacking. In our study, a statistically significant history of coincident triggering factor with vitiligo was elicited commonly in those with positive anti-TPO Ab status as compared to those with anti-TPO Ab negative status. A suitable reason for the association of trigger factors being commoner in those participants with anti-TPO Ab positivity than those without could not be ascertained and probably a larger study over a longer duration of time measuring association of thyroid autoimmunity with individual precipitating factors in vitiligo would be able to determine roles played by trigger factors in vitiligo and associated thyroid autoimmunity in vitiligo patients. In the lack of robust evidence, fictitious differences attributable to recall bias cannot be ruled out. Moreover, our study did not formalize definitions for “triggers” and probing undertaken by the researcher on each participant was not standardized and was guided by clinical acumen.

#### 5.3.6. Disease Activity in the Participants

A low VIDA score indicates less activity. In our study, (19.4%) of the anti-TPO Ab negative participants had a VIDA score of “0” or “−1” as compared to only 3.6% of those found positive. All but one (96.4%) of the anti-TPO Ab positive participants reported disease activity with a VIDA score “≥1” indicating that anti-TPO Ab positivity could be associated with progressive vitiligo disease activity. This could also suggest that majority of our cases could be of type A or autoimmune vitiligo which is known to progress continuously with alternate periods of remissions and exacerbations [32].

#### 5.3.7. Type of Vitiligo and Clinical Variants

Vitiligo vulgaris was the commonest type of vitiligo in our study irrespective of anti-TPO Ab status. In an Indian study by Dave and colleagues, mucosal vitiligo was found to be significantly associated with thyroid autoimmunity but in our study no significant difference was found in the representation of segmental and nonsegmental vitiligo in the two groups [9]. None of the 50 patients with segmental vitiligo showed any thyroid dysfunction (*P* = 0.047) in the study by Cho et al. [33].

#### 5.3.8. Site of Onset of Vitiligo

With regard to the site of onset, while an equal number of participants in the anti-TPO Ab positive group reported an onset of vitiligo in head and neck, trunk, upper limb, and lower limb (25% each), none reported genitals as a site of onset. In the anti-TPO Ab negative group, majority had onset of vitiligo in the lower limb (37.5%) followed by head and neck and trunk regions (20.8% each). We did not find significant association of site of onset with anti-TPO Ab status which is in accordance with other studies that have evaluated the same [32].

#### 5.3.9. Vitiligo Area Severity Index (VASI) [25]

The mean VASI percentage in the anti-TPO positive group was 2.40% and anti-TPO negative was 3.12% but it did not have a significant difference (*P* = 0.376). We could not find other studies which have compared VASI with anti-TPO Ab, but a significant association of greater body surface area involved (calculated by rule of nines) with positive thyroid antibodies was reported by Nunes and Esser [28].

#### 5.3.10. Clinical Variants

While nearly 10% of the participants with anti-TPO Ab positivity had the trichrome variant of vitiligo, the proportion was just 4.2% for their anti-TPO Ab negative counterparts. Similarly around 7% of the participants with anti-TPO Ab positivity had the quadrichrome variant of vitiligo; the proportion was 5.2% for their anti-TPO Ab negative counterparts. Majority (82.1% and 90.3% participants in these respective groups) could not be identified with any particular variant.

#### 5.3.11. Clinical Characteristics of Vitiligo Lesion

Even as we obtained a *P* value of 0.132, we note that koebnerization was more frequently reported in anti-TPO Ab positive participants (10.7%) as compared to the anti-TPO Ab negative ones (2.8%). Leukotrichia was equally frequent in both groups (around 86% in both). Halo nevus was present in only one patient who was anti-TPO Ab negative. Ezzedine et al. noted the presence of halo naevi, circulating antithyroid antibodies and leukotrichia more often in patients with mixed vitiligo (65.4%, 23.1%, and 92.0%, resp.) than those with segmental vitiligo (12.0%, 5.9%, and 36.0%, resp.). Thus, they inferred halo naevi association and leukotrichia at first consultation in segmental vitiligo as risk factors for the progression of segmental vitiligo to mixed vitiligo [34].

#### 5.3.12. Association of Anti-TPO Ab with Thyroid Function Status

The serum TSH levels in either group showed a skewed distribution with no significant difference (*P* = 0.514). However, the mean TSH level was almost five times higher in anti-TPO Ab positive participants than the anti-TPO Ab negative subjects. We explored if serum TSH levels had a correlation with the anti-TPO Ab antibody titre. A correlation coefficient of 0.052 disproved this and highlighted that these are probably mutually unrelated (*P* = 0.609). Previously Imam et al. have noted a positive significant correlation between serum anti-TPO A band serum TSH [35]. In the present study, most of the participants in either of the categories reported a euthyroid status. Only two (7.1%) of the anti-TPO Ab positive participants and one (1.4%) of the negative participants had hyperthyroidism. Similarly, only five (17.9%) in the former and eight (11.1%) in the latter were hypothyroid. We observed that no significant association was present between the anti-TPO antibody status and the thyroid function status (euthyroid versus thyroid dysfunction) of the study participants (*P* = 0.126). An Indian study by Dave and colleagues reported that biochemical abnormality (predominantly hyperthyroidism) was found in 40% of the cases as against 6.7% of the controls (*P* < 0.05) [9].

#### 5.3.13. Association with Other Diseases

We did not find any significant association of coexistent autoimmune, dermatological, or systemic diseases with anti-TPO Ab status of the study participants. 

*Limitations*. We have discussed the implicated study limitations under relevant subsections. One overriding limitation of this study is the use of a sole marker (anti-TPO Ab) for identifying thyroid autoimmunity in the participants. Both antithyroglobulin and anti-TPO antibodies are valuable for screening thyroid autoimmunity. Any comprehensive study in this context should employ both tests. However, due to resource constraints we had to make the tough decision of choosing any of them. Upon the literature review we found that antithyroperoxidase antibodies are a better screening tool than antithyroglobulin antibodies. The former has better sensitivity and correlation with thyroid autoimmunity as compared to the latter [36, 37]. Biochemical assays for this study were undertaken in the institution's central laboratory. We have assumed that the laboratory results are consistent and standardized. One of the strengths of this study was that the researcher was blinded to the anti-TPO status of the participant at the time of the data collection interview. Known thyroid patients were excluded from the study for this reason. This allowed for nonbiased collection of information from the participants.

## 6. Conclusion

The prevalence of thyroid autoimmunity in vitiligo patients (anti-TPO antibodies serum titre ≥35 IU/mL) at the study site was found to be 28% (19.2–36.8%). We found a significant relationship of anti-TPO Ab with a higher mean age of the patients with majority being in their third decade and beyond. Patients reporting coincident precipitating trigger for vitiligo lesions were more likely to report positive for anti-TPO Ab.

In their study, Mandry et al. noted that 14% of patients with concomitant vitiligo and thyroid autoantibody positivity showed presence of overt autoimmune/endocrine disease implying the high incidence of latent disease which would probably manifest only during follow-up [13]. This is in conformity with findings of other studies that vitiligo often precedes thyroid dysfunction. Hence, screening vitiligo patients for thyroid autoimmunity so as to diagnose and initiate treatment for any comorbidity with the anti-TPO Ab titre seems to be relevant as this antibody is a sensitive and specific marker of autoimmune thyroid disorders. This is especially recommended in older patients, more so in female patients, and those reporting a history of a precipitating factor and or history of longer duration. Perhaps, studies on a larger number of vitiligo patients with control group from general population who can be followed up for a considerable time would further strengthen these associations and also identify other specific clinical parameters of vitiligo that could raise suspicion for associated thyroid autoimmunity.

## Figures and Tables

**Table 1:** Sample characteristics of study participants.

Sl. number	Characteristic variable	Total (*N* = 100)
1	Number of male, female participants	41, 59
2	Age^#^ (years)	29.49 ± 15.00; 28.00
3	Reported age at onset of vitiligo^#^ (years)	24.80 ± 15.66; 21.25
4	Duration since first lesion of vitiligo^#^ (years)	4.64 ± 6.05; 2.00
5	Positive history of vitiligo in family (%)	13.00
6	Positive history of precipitating factors (%)	8.00
7	Stable vitiligo [Vitiligo Disease Activity (VIDA) Score ≤ 0]	15
8	Type of vitiligo (segmental; nonsegmental) (%)	4.00; 96.00
9	Vitiligo Assessment Severity Index (VASI) <1.00 (%)	22.00
10	Variants of vitiligo (quadrichrome and trichrome) (%)	12.00
11	Koebner's phenomenon present (%)	05.00
12	Leukotrichia present (%)	14.00
13	Associated dermatological disorders (%)	5.00
14	Associated systemic disorder (%)	4.00^$^
15	Associated nonthyroid autoimmune disorder (%)	2.00^@^

^#^Mean ± standard deviation; median; ^$^all reported diabetes mellitus; one reported coexistent bronchial asthma; ^@^one participant reported alopecia areata and duodenitis/gastritis with pernicious anaemia.

**Table 2:** Sample characteristics of study participants according to anti-TPO Ab status.

		Anti-TPO Ab status		
Sl. number	Characteristic variable	Positive	Negative	*P* value
		(>35 IU/mL)	(<35 IU/mL)	
1	Number of participants	28	72	—
2	Number of male, female participants	8, 20	33, 39	0.115
3	Age^#^	34.50 ± 13.99	27.54 ± 15.02	**0.037** ^*^
4	Reported age at onset of vitiligo^*^	24.59 ± 15.46	24.88 ± 15.83	0.982
5	Duration since first lesion of vitiligo^*^	5.19 ± 6.63	4.42 ± 5.85	0.700
6	Positive history of vitiligo in family (%)	10.71	13.89	1.000
7	Positive history of precipitating factors (%)	17.86	4.17	**0.037** ^*^
8	Stable vitiligo [VIDA Score ≤ 0]	3.57	19.44	0.222
9	Vitiligo type (segmental) (%)	3.57	4.17	1.000
10	VASI <1.00 (%)	32.14	18.06	0.376
11	Vitiligo variants (quadrichrome and trichrome) (%)	17.86	9.72	0.308
12	Koebner's phenomenon present (%)	10.71	2.78	0.132
13	Leukotrichia present (%)	14.29	13.89	1.000
14	Associated dermatological disorders (%)	7.14	04.17	0.617
15	Associated systemic disorder (%)	0	5.56	0.574
16	Associated nonthyroid autoimmune disorder (%)	7.14	0	0.076
17	Serum TSH titres (*μ*IU/mL)	11.31 ± 41.03	2.67 ± 2.27	0.514
18	Euthyroid (%)	75.00	87.50	0.139

^#^Mean ± standard deviation; median in years.

^*^Statistically significant (*P* < 0.05).

**Table 3:** Comparison of thyroid autoantibody profile in various studies.

Sl. number	Studies	Reported prevalence (%) of anti-TPO Ab in vitiligo patients
1	Cunliffe et al. (1968) [5]	11.6
2	Betterle et al. (1985) [10]	18.4
3	Schallreuter et al. (1994) [11]	10.7
4	Hegedus et al. (1994) [12]	25.2
5	Mandry et al. (1996) [13]	50.0
6	Dave et al. (2003) [9]	17.1
7	Daneshpazhooh et al. (2006) [14]	18.1
8	Altaf et al. (2010) [15]	11.0
9	Our study in discussion (2013)	28.0 (19.2–36.8)
